# Supplementary figures and images for: Meiotic double-strand break repair DNA synthesis tracts in Arabidopsis thaliana
Source: PLoS Genet. 2024 Jul 16;20(7):e1011197. doi: 10.1371/journal.pgen.1011197 (PMC11280534; doi:10.1371/journal.pgen.1011197)

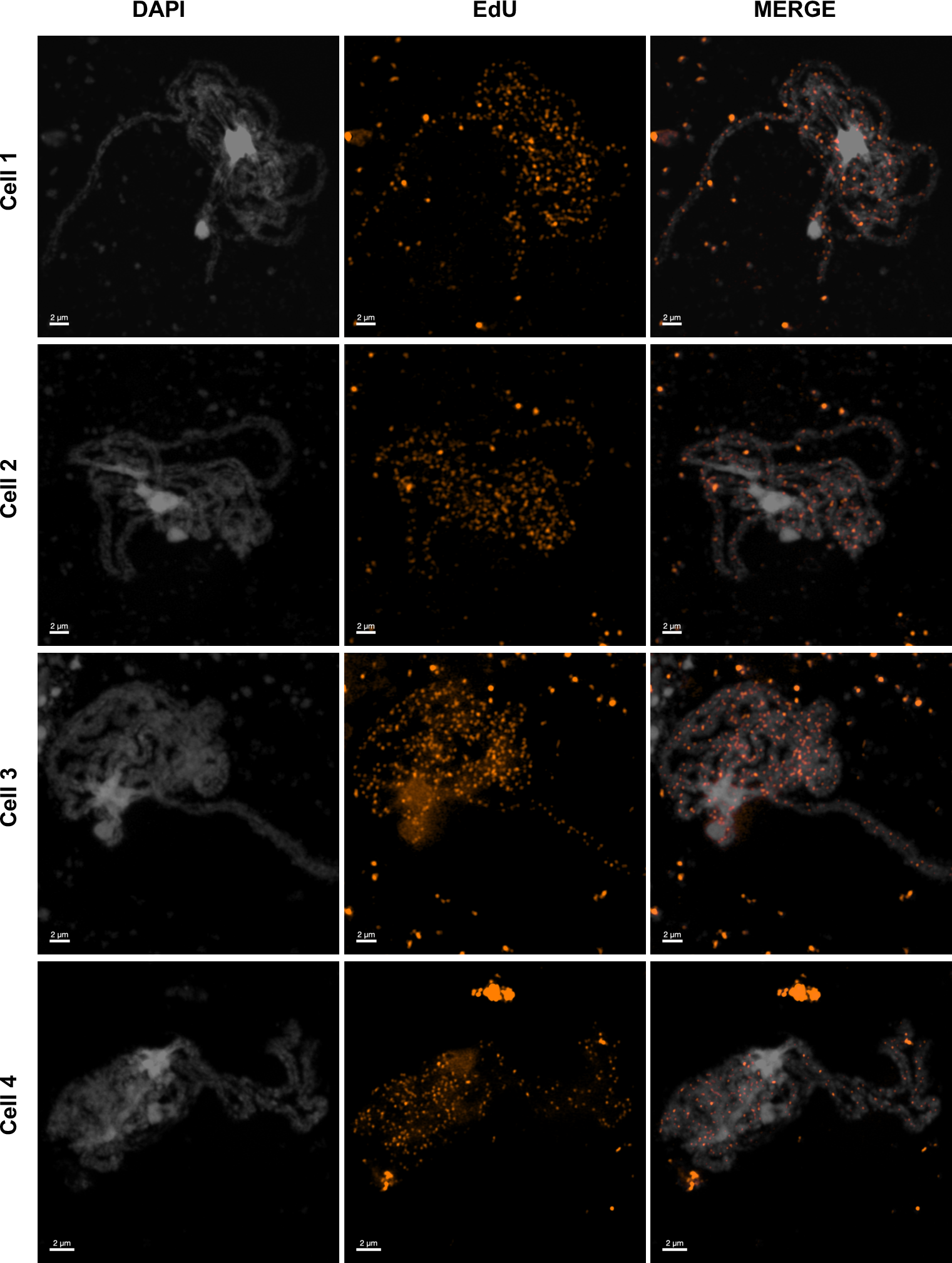

Supplement: S1 Fig — Examples of prophase I DNA labelling in pachytene nuclei of wildtype PMC. Each row is the same nucleus, imaged with DAPI fluorescence (white, left), EdU (orange, middle) and the merged image (right). Images taken with the confocal microscope with Airyscan module. 2μm scale bars are included at the bottom left of each image. (TIF) [file pgen.1011197.s001.tif]

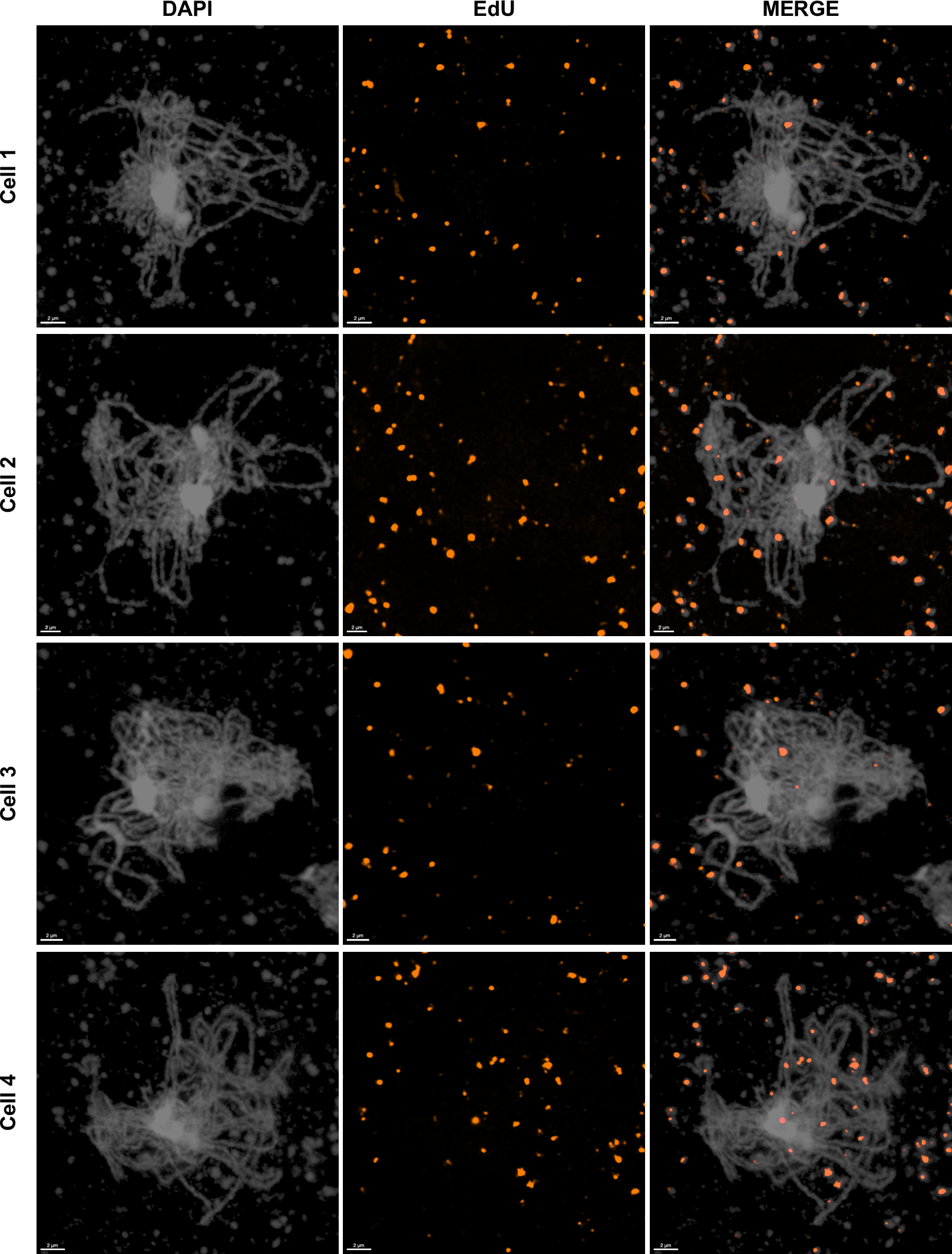

Supplement: S2 Fig — Examples of prophase I DNA labelling in mid-prophase nuclei of spo11-1 PMC. Each row is the same nucleus, imaged with DAPI fluorescence (white, left), EdU (orange, middle) and the merged image (right). Images taken with the confocal microscope with Airyscan module. 2μm scale bars are included at the bottom left of each image. (TIF) [file pgen.1011197.s002.tif]

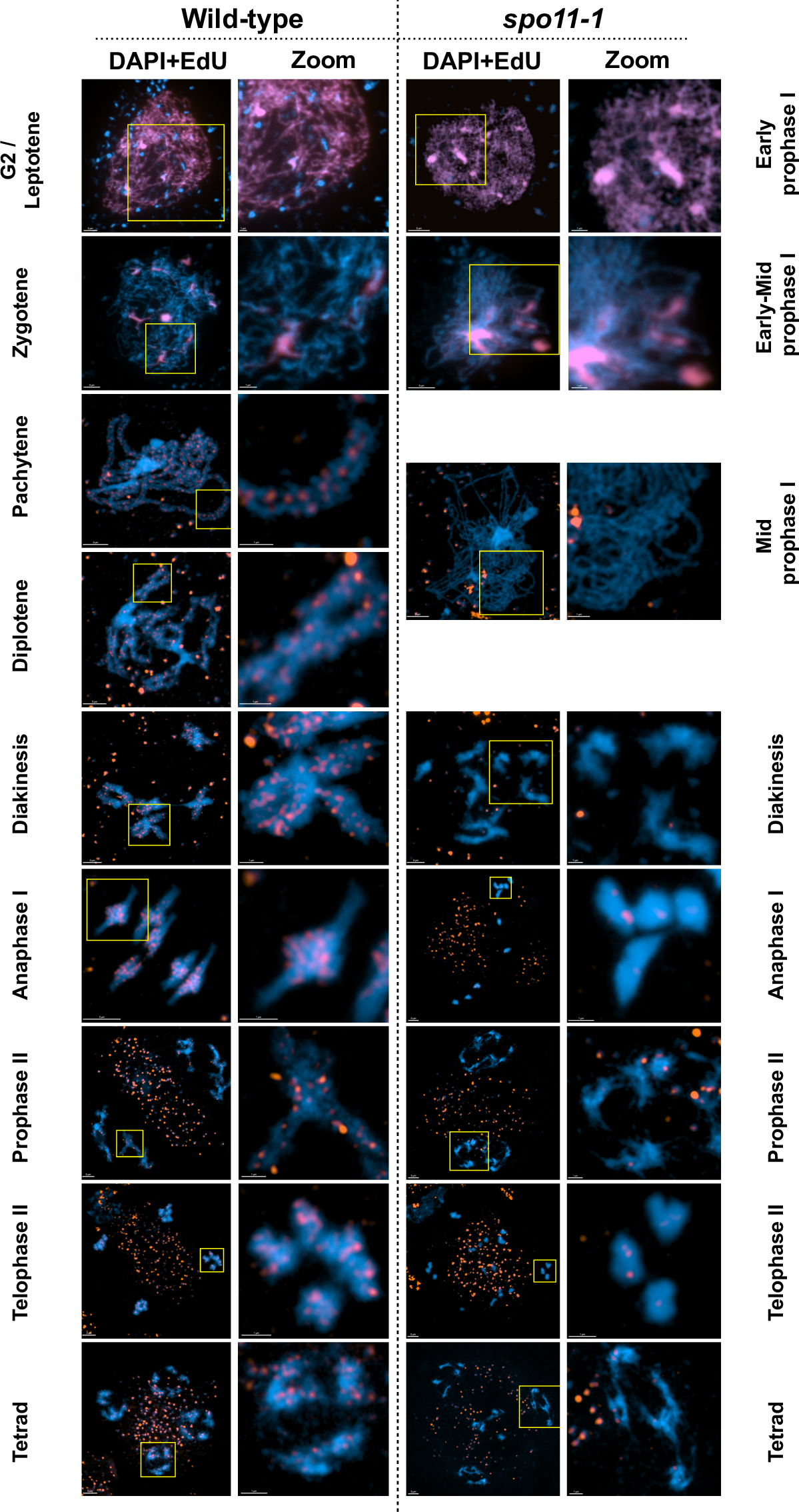

Supplement: S3 Fig — DAPI+EdU images of meiotic stages taken from Fig 2 (Wild-type, left two columns) and Fig 3 (spo11-1, right two columns), aligned to facilitate comparison. 3μm scale bars are shown at the bottom left of each image (1μm for the zooms) and rectangles in columns 1 and 3 indicate the corresponding enlarged regions presented in the zooms in columns 2 and 4. (TIF) [file pgen.1011197.s003.tif]

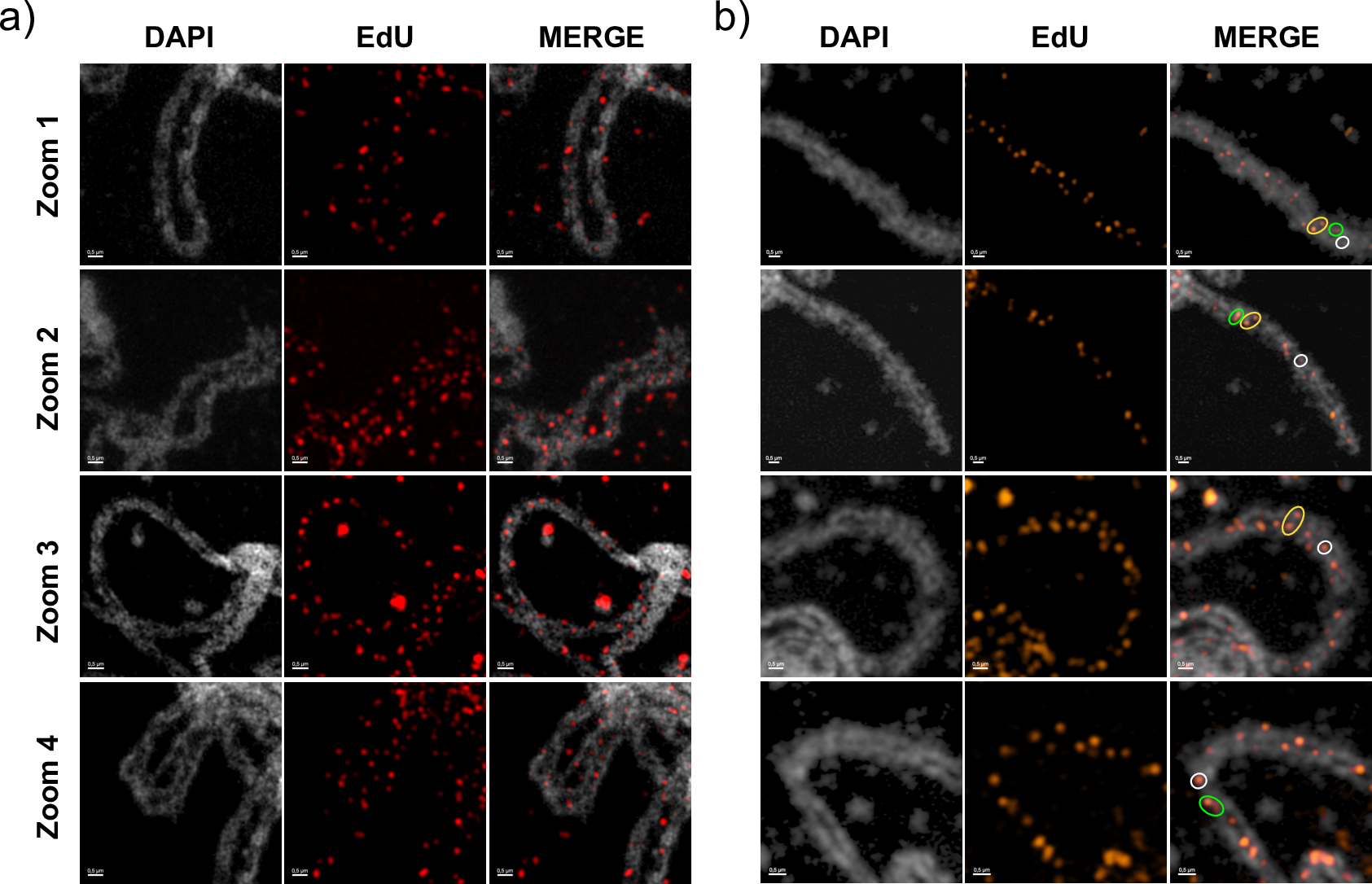

Supplement: S4 Fig — Zoomed regions from SIM (a) and Confocal (b) images of meiotic pachytenes, showing the distribution of EdU-labelled meiotic prophase I DNA synthesis foci (a)red, b) orange) on the DAPI-stained chromosome fibres (white) of the synaptonemal complexes. Examples of isolated individual foci (one SC lateral axis, white circle) and pairs of foci on one homologue (one SC lateral axis, green circle) or the two homologues (both SC lateral axes, yellow circle) are highlighted. 0.5μm scale bars are included at the bottom left of each image. (TIF) [file pgen.1011197.s004.tif]
